# Regulatory review of benefits and risks of preventing infant RSV disease through maternal immunization

**DOI:** 10.1038/s41541-024-01002-y

**Published:** 2024-10-31

**Authors:** Yugenia K. Hong-Nguyen, Joseph Toerner, Lucia Lee, Maria C. Allende, David C. Kaslow

**Affiliations:** 1 U.S. Food and Drug Administration (FDA), 10903 New Hampshire Avenue, Silver Spring, MD 20903 USA; 2 Center for Biologics Evaluation and Research (CBER), 10903 New Hampshire Avenue, Silver Spring, MD 20903 USA; 3 Office of Vaccines Research and Review (OVRR), 10903 New Hampshire Avenue, Silver Spring, MD 20903 USA; 4 Division of Clinical and Toxicology Review (DCTR), 10903 New Hampshire Avenue, Silver Spring, MD 20903 USA; 5 Chase Brexton Health Care, 5500 Knoll North Dr. #370, Columbia, MD 21045 USA; 6 Center for Drug Evaluation and Research (CDER), 10903 New Hampshire Avenue, Silver Spring, MD 20903 USA; 7 Office of Immunology and Inflammation (OII), 10903 New Hampshire Avenue, Silver Spring, MD 20903 USA; 8 Division of Hepatology and Nutrition (DHN), 10903 New Hampshire Avenue, Silver Spring, MD 20903 USA; 9 Immediate Office of the Director, 10903 New Hampshire Avenue, Silver Spring, MD 20903 USA

**Keywords:** Drug regulation, Drug development

## Abstract

In August 2023, FDA approved Abrysvo for active immunization of pregnant individuals at 32 through 36 weeks gestational age to prevent lower respiratory tract disease (LRTD), including severe LRTD, caused by respiratory syncytial virus (RSV) in infants from birth through 6 months of age. A pragmatic approach to narrow the interval of use of Abrysvo in pregnant individuals balanced benefits of vaccine effectiveness against potential risks to infant and mother.

Recently, the United States Food and Drug Administration (FDA) approved a vaccine for active immunization of pregnant individuals to prevent lower respiratory tract disease (LRTD), including severe LRTD, caused by respiratory syncytial virus (RSV) in their infants from birth through 6 months of age. Herein the authors provide additional perspective regarding regulatory review of the Biologics License Application (BLA) of Respiratory Syncytial Virus Vaccine (Abrysvo). We describe a pragmatic approach taken to narrow the interval of vaccine use in pregnant individuals to 32 through 36 weeks gestational age, thereby avoiding the potential risk of very preterm or extremely preterm births with vaccine use before 32 weeks of gestation.

RSV is a common cause of bronchiolitis and viral pneumonia in infants and the leading cause of infant hospitalizations in the U.S^1^. In considering the public health impact of preventing RSV LRTD in infants by active immunization of pregnant individuals, it is important to note that: (1) the highest rates of RSV hospitalization occur within the first 3 months of life; and (2) in hospitalized infants, RSV disease is associated with a 1–3% fatality rate. Risk factors for severe disease include prematurity, underlying chronic lung or heart disease, and immunodeficiency; however, even healthy infants through 6 months of age are at significant risk for morbidity and mortality^2^. In the 1960s, an investigational formalin-inactivated RSV vaccine studied in RSV-naive infants and toddlers was associated with enhanced respiratory disease (ERD) following natural infection with RSV^3^. Greater understanding of potential immunopathologic ERD mechanisms^4^ and advances in understanding the structural immunology of RSV surface fusion (F) glycoprotein^5^ facilitated subsequent RSV vaccine development.

Development of RSV stabilized prefusion F subunit (RSVPreF) vaccines for prevention of RSV disease came to fruition with BLA submissions to FDA in late 2022. In Spring 2023, FDA approved two recombinant RSV prefusion F subunit vaccines, Arexvy (Respiratory Syncytial Virus Vaccine, Adjuvanted) and Abrysvo (Respiratory Syncytial Virus Vaccine), for active immunization of individuals 60 years of age and older for the prevention of LRTD caused by RSV. On August 21, 2023, FDA approved Abrysvo for active immunization of pregnant individuals at 32 through 36 weeks gestational age for the prevention of LRTD, including severe LRTD, caused by RSV in infants from birth through 6 months of age^6^.

While both Abrysvo and RSVPreF3-Mat (an unadjuvanted RSV Prefusion F Protein-Based Maternal Vaccine) were being developed for active immunization of pregnant individuals for the prevention of RSV disease in infants, clinical development of RSVPreF3-Mat was halted during a prespecified interim safety analysis in 2022. An imbalance in the percentage of preterm births and neonatal deaths was observed between the vaccine and the placebo groups. The difference in the percentage of neonatal deaths between the RSVPreF3-Mat and placebo groups was likely attributable to the imbalance in preterm births and extremes of prematurity^7^. To date, an explanation has not been reported for the apparent increased rate of preterm births observed following vaccination with RSVPreF3-Mat.

Alternative therapeutics to active immunization during pregnancy for prevention of RSV disease in infants include two RSV F protein inhibitor monoclonal antibodies that FDA approved for prevention of LRTD caused by RSV in infants and toddlers. FDA approved nirsevimab-alip injection on July 17, 2023 for prevention of LRTD caused by RSV from birth during a first RSV season and in children up to 24 months of age who remain vulnerable to severe RSV disease^8^. Palivizumab injection was approved by FDA in 1998 for the prevention of serious LRTD caused by RSV in children at high risk of RSV disease.

A Phase 3, randomized, double-blind, multicenter, placebo-controlled study (NCT04424316) assessed the efficacy of Abrysvo in the prevention of RSV-associated LRTD in infants born to individuals vaccinated during pregnancy. Vaccine efficacy (VE) against severe LRTD due to RSV was established at all prespecified time points, including VE of 69.4% (97.6% CI 44.3%, 84.1%) in infants within 180 days after birth. VE for any LRTD due to RSV was 51.3% (97.6% CI 29.4%, 66.8%) in infants within 180 days after birth. A prespecified secondary endpoint also showed a statistically significant reduction in infant hospitalization due to RSV LRTD. In the subgroup of pregnant individuals vaccinated at 32 through 36 weeks gestational age, the point estimate of vaccine efficacy for prevention of severe LRTD due to RSV in infants was 76.5% (95% CI 41.3%, 92.1%) within 180 days after birth, while vaccine efficacy for preventing any LRTD due to RSV in infants was 57.3% (95% CI 29.8%, 74.7%) within 180 days after birth, mirroring the efficacy findings in the overall trial population.

FDA’s evaluation of the safety data submitted in the Abrysvo BLA (which included a safety population of 7357 maternal participants; 3681 in the Abrysvo group and 3676 in the placebo group, as of the safety data cutoff date of September 2, 2022) revealed a numerical imbalance of preterm births in pregnant individuals following Abrysvo vaccination in both the phase 3 trial and a smaller phase 2 trial. FDA considered this imbalance when assessing the benefits and risks for immunization of pregnant individuals at 24 through 36 weeks of gestational age for prevention of LRTD caused by RSV in their infants. Specifically, FDA was concerned that the observed 1% difference in preterm births (i.e., 5.7% among Abrysvo recipients and 4.7% among placebo recipients in the phase 3 trial) might represent a true vaccine-associated risk of preterm birth.

Given this concern, FDA performed a comprehensive evaluation of the available safety data for preterm births in the phase 3 trial of Abrysvo administered to pregnant individuals at 24 through 36 weeks gestational age. Preterm birth was defined as birth occurring before 37 weeks of gestation. In the overall study population, preterm birth was reported in 5.7% of infants (202/3568, 95% CI 4.9%, 6.5%) in the Abrysvo group and 4.7% (169/3558, 95% CI 4.1%, 5.5%) in the placebo group. Thirty-three (33) preterm births occurred before 34 weeks gestation (21 in the vaccinated group and 12 in the placebo group), of which 1 infant in each study group was born extremely preterm (defined as <28 weeks gestation). Approximately 90% of preterm births in the overall trial population occurred between 34 to <37 weeks of gestational age.

Of infants born to pregnant individuals administered study vaccine/placebo at 32 through 36 weeks gestational age, 4.2% (68/1631) in the Abrysvo group and 3.7% (59/1610) in the placebo group were born preterm. In this subgroup of pregnant individuals administered study vaccine/placebo at 32 through 36 weeks gestational age (45% of the trial population), 2 infants in each study group were born before 34 weeks of gestation. The imbalance in preterm births was 0.5%.

Prematurity overall did not appear to be temporally associated with vaccination, with the majority of participants delivering outside of the 30-day AE reporting period. An analysis of the time from vaccination to the time of preterm delivery did not show a difference between the Abrysvo group and the placebo group. Although the median time from vaccination to preterm delivery varied between subgroups by gestational age, overall the median time from vaccination to preterm delivery was approximately 40 days (see Clinical Review Memo – ABRYSVO^9^). Available data were insufficient to establish or exclude a causal relationship between preterm birth and administration of Abrysvo. Similarly, a mechanistic hypothesis has yet to be identified that might provide a plausible causal explanation for preterm birth risk.

The imbalance in hypertensive disorders of pregnancy (HDP) observed in vaccinated pregnant individuals raised an additional safety concern. Pregnancy-associated serious adverse events (SAEs) that could prompt earlier delivery for maternal or fetal indications (e.g., HDP, premature rupture of membranes, and preterm premature of membranes) were reported in 4.1% in the Abrysvo group and 3.3% in the placebo group; this included preeclampsia in 1.8% of participants in the Abrysvo group and 1.4% in the placebo group. The incidence rates of preterm births and pregnancy-associated SAEs in the clinical development of Abrysvo were lower than the expected rates in the general population; however, FDA noted that pregnant individuals selected for clinical trial enrollment were generally a healthier population at lower risk for pregnancy complications.

FDA’s decision to approve Abrysvo for the indication of active immunization of pregnant individuals at 32 through 36 weeks of gestational age for the prevention of LRTD and severe LRTD caused by RSV in infants from birth through 6 months of age used a pragmatic approach to the evaluation of risks and benefits. That is, the overall findings of vaccine efficacy in the phase 3 trial in pregnant individuals met the Agency’s statutory requirements for establishing effectiveness by one trial with supporting data from a phase 2 trial in pregnant individuals as well as the established safety profile and effectiveness of Abrysvo in the older adult population. Narrowing the timing of immunization in pregnant individuals to 32 through 36 weeks gestational age avoids the potential risk of preterm birth before 32 weeks of gestational age, which includes very preterm or extremely preterm births, if the risk of preterm births represents a true risk. Analyses of subgroups for efficacy and safety provided support to narrow the interval of vaccine use to 32 through 36 weeks gestational age. FDA required four postmarketing safety studies to further evaluate the potential risks of preterm birth and HDP. The current Prescribing Information provides adequate risk mitigation by informing health care professionals of the benefits and risks associated with vaccination to adequately assess its implications for prescribing decisions in individual patient management.

Approval of Abrysvo for this narrower interval of vaccine use allowed for a favorable balance of benefits of vaccine effectiveness against potential risks of preterm birth and HDP, with thorough, informative Prescribing Information for health care providers. As with other approved vaccines, FDA is using postmarketing studies and established safety surveillance programs to carefully monitor for new and additional safety information for Abrysvo.
