# Author Correction: Regulatory review of benefits and risks of preventing infant RSV disease through maternal immunization

**DOI:** 10.1038/s41541-025-01092-2

**Published:** 2025-03-05

**Authors:** Yugenia K. Hong-Nguyen, Joseph Toerner, Lucia Lee, Maria C. Allende, David C. Kaslow

**Affiliations:** 1 U.S. Food and Drug Administration (FDA), 10903 New Hampshire Avenue, Silver Spring, MD 20903 USA; 2 Center for Biologics Evaluation and Research (CBER), 10903 New Hampshire Avenue, Silver Spring, MD 20903 USA; 3 Office of Vaccines Research and Review (OVRR), 10903 New Hampshire Avenue, Silver Spring, MD 20903 USA; 4 Division of Clinical and Toxicology Review (DCTR), 10903 New Hampshire Avenue, Silver Spring, MD 20903 USA; 5 Chase Brexton Health Care, 5500 Knoll North Dr. #370, Columbia, MD 21045 USA; 6 Center for Drug Evaluation and Research (CDER), 10903 New Hampshire Avenue, Silver Spring, MD 20903 USA; 7 Office of Immunology and Inflammation (OII), 10903 New Hampshire Avenue, Silver Spring, MD 20903 USA; 8 Division of Hepatology and Nutrition (DHN), 10903 New Hampshire Avenue, Silver Spring, MD 20903 USA; 9 Immediate Office of the Director, 10903 New Hampshire Avenue, Silver Spring, MD 20903 USA

**Keywords:** Drug regulation, Drug development

Correction to: *npj Vaccines* 10.1038/s41541-024-01002-y, published online 31 October 2024

The original version of this Article stated that Arexvy rather than the non-adjuvanted RSVPreF3-Mat was used in GlaxoSmithKline Pharmaceuticals (GSK’s) maternal immunization clinical program. Therefore, the Article has been corrected with reference to “RSVPreF3-Mat” and the removal of “Arexvy” in the context of discussing GSK’s maternal immunization program.

Additionally, the original version of this Article stated, “A higher number of neonatal deaths associated with extremely preterm births was observed among pregnant individuals who received Arexvy [RSVPreF3-Mat].” It is noted that while the difference in preterm births between the RSVpreF3-Mat and placebo groups in this trial was a statistically significant finding, the imbalance in neonatal deaths was not statistically significant and was likely attributable to the imbalance in preterm births, as reported in Dieussaert, I. et al. (N. Engl. J. Med. 390, 1009–1021 [2024]). It should also be noted that approaches to evaluation of the safety of a drug or vaccine differ from methods used to evaluate effectiveness. Statistical significance need not be a major determinant in the evaluation of safety in the premarketing setting. Therefore, this Article has been revised to describe the “imbalance” in the percentages of preterm births and neonatal deaths between the RSVpreF3-Mat group and placebo group.

These corrections do not change the scientific conclusions of the article. The original article has been corrected.

